# The Relativistic Car: Applying Metaethics to the Debate about Self-Driving Vehicles

**DOI:** 10.1007/s10677-021-10190-8

**Published:** 2021-05-22

**Authors:** Thomas Pölzler

**Affiliations:** Department of Philosophy, University of Graz, Attemsgasse 25/II, Graz, Austria

**Keywords:** Self-driving cars, Artificial intelligence, Metaethics, Moral relativism, Moral universalism

## Abstract

Almost all participants in the debate about the ethics of accidents with self-driving cars have so far assumed moral universalism. However, universalism may be philosophically more controversial than is commonly thought, and may lead to undesirable results in terms of non-moral consequences and feasibility. There thus seems to be a need to also start considering what I refer to as the “relativistic car” — a car that is programmed under the assumption that what is morally right, wrong, good, bad, etc. is determined by the moral beliefs of one’s society or culture. My investigation of this idea involves six steps. First, I explain why and how the moral universalism/relativism debate is relevant to the issue of self-driving cars. Second, I argue that there are good reasons to consider accident algorithms that assume relativism. Third, I outline how a relativistic car would be programmed to behave. Fourth, I address what advantages such a car would have, both in terms of its non-moral consequences and feasibility. Fifth, I address the relativistic car’s disadvantages. Finally, I qualify and conclude my considerations.

## Introduction

In some years or decades self-driving cars^1^ will be a common sight on the roads of many countries. These cars will be (and have already been) involved in accidents whose outcomes depend on how they are programmed. Based on some algorithms different people will be harmed or put to risk than based on others. This raises an important moral question. How ought self-driving cars to be programmed to behave in such situations (Nyholm 2018a, 2018b)?

To illustrate this question, consider the following scenario (taken from Awad et al. 2018). A self-driving car suffers from a sudden break failure. If it stays on course it will kill a boy who crosses the street despite a “do not cross” signal. If it swerves to the right it will kill an adult who is crossing the street on a “go ahead” signal. Which of these options ought the self-driving car be programmed to take? That is, should it kill the boy or the adult?^2^

So far philosophers have mainly addressed such questions by importing or applying tools from normative ethics. For example, they have appealed to theories such as consequentialism, contractualism or deontology (e.g., Bonnefon et al. 2015; Leben 2017), and have drawn inferences from the literature about trolley cases (e.g., Lin, 2015; Wallach and Allen, 2009; for discussion see Hübner and White 2018; Nyholm and Smids 2016). My focus in this paper, by contrast, will be on the implications of *metaethical* claims, i.e., dominantly descriptive philosophical claims about morality (Miller 2003; Huemer 2005). More specifically, I attempt to inform the ethics of accidents with self-driving cars by considering the universalism/relativism debate about the scope of moral judgements (Gowans 2015).^3^

Almost all participants of the debate about the ethics of accidents with self-driving cars have so far tacitly assumed moral universalism. That is, they have assumed that — at least when it comes to this particular moral matter — there is a single true morality that applies to all individuals and groups, regardless of their beliefs, traditions, practices, sentiments, etc. (see, e.g., Bonnefon et al. 2015; Gerdes and Thornton 2015 for a general endorsement of a consequentialist framework, and Leben 2017 for a general endorsement of a Rawlsian framework).^4^ But moral universalism may be philosophically more controversial than is commonly thought (see Sec. 2), and it may also lead to undesirable results in terms of non-moral consequences and feasibility (see Sec. 4). There thus seems to be a need to also start considering what I will henceforth refer to as a “relativistic car” — a car that is programmed under the assumption of the truth of moral relativism.

Moral relativism is the view that the truth or falsity of moral judgements depends on the beliefs, traditions, practices, sentiments etc. of individuals or groups. In what follows I will focus on a simple generic version of this view (see Gowans 2015). However, many of the paper’s insights will also extend to more complex versions that have actually been defended (e.g., Harman 1996; Prinz 2007; Velleman 2013). According to moral relativism in the generic sense that is addressed here, moral truth and falsity are relative to groups, in particular to large groups such as *societies or cultures* (as opposed to individuals or small groups); they are relative to these groups’ *moral beliefs* (as opposed to their traditions, practices, sentiments, etc.); and, more precisely, they are relative to the moral beliefs of those who make moral judgements about the matter at issue, i.e., to the *appraisers* (as opposed to those individuals or groups about whom judgements are made, i.e., the agents).

My investigation of the relevance and implications of moral relativism for the ethics of accidents with self-driving cars will be preliminary; it will first and foremost aim at raising awareness for the issue and motivating further (more detailed) studies. I will begin by explaining why and how the moral universalism/relativism debate is relevant to the issue of self-driving cars (Sec. 1). Then I will argue that there are good reasons to not only consider accident algorithms that assume universalism, but also those that assume relativism (Sec. 2). I will outline how a relativistic car would be programmed to behave (Sec. 3); and last but not least, I will address the advantages that such a car would have, both in terms of its non-moral consequences and feasibility (Sec. 4), as well as its disadvantages (Sec. 5).

## The Relevance of the Universalism/Relativism Debate

Universalism and relativism are claims in ethics; more specifically, in metaethics.^5^ This may lead one to wonder whether they are practically relevant for the issue of accidents with self-driving cars. Isn’t it the same, in terms of how these cars ought to be programmed, whether moral judgements apply universally or only relatively? And haven’t ethicists thus been right to largely ignore the universalism/relativism debate in this context?

A first potential reason for thinking so applies to ethical considerations about self-driving cars in general. These cars will be programmed to conform to the laws of the countries in which they operate. For example, if a car is produced in the US, exemplars that are intended for the Japanese market will conform to Japanese (rather than to US) law. Add to this that traffic is a tightly regulated domain, with laws for speeding, right of way, etc. determining the overwhelming majority of decisions, and it appears that moral judgements will be of very little consequence. For example, whether self-driving cars ought to kill the boy or the adult in the above example appears to be a matter of law rather than of morality.^6^

This objection, though involving some truth, exaggerates the extent to which traffic decisions are and will be covered by laws, especially in the transition period from ordinary to self-driving cars (in which the law will continuously have to catch up with technological developments). For example, even though in the US city of Phoenix “rider-only” cars have already been running for more than a year,^7^ there is not yet any law which determines whether in cases of unavoidable accidents these cars ought to kill a boy who crosses the street despite a “do not cross” signal or an adult who crosses the street on a “go ahead” signal. In light of the myriads of different situations that can arise in traffic a large number of cars’ decisions will always remain underdetermined by the law and are hence subject to moral considerations that are practically relevant.

Even if one accepts that ethics in general is relevant to programming self-driving cars, one might still deny that this holds for the particular metaethical debate between moral universalism and relativism. The most natural objection to this effect starts from the observation that moral universalism can be combined with some version of a principle of tolerance (e.g., Brink 1989). Tolerant universalists can hold that self-driving cars ought to kill the adult who is crossing the street on a “go ahead” signal in all societies and cultures, even in societies and cultures that dominantly favor killing the boy, whilst also holding that it would be wrong to try to convert or interfere with the behavior of these latter societies and cultures. Hence, this kind of universalism discourages (at least some or most) attempts of making accident algorithms in morally divergent societies or cultures conform to those of one’s own.

Universalism’s and relativism’s relationship to tolerance has been subject to continuous metaethical debate (see Gowans 2015). I agree that universalism can (and should) be combined with a principle of tolerance. However, just as relativism, it does not by itself entail or suggest such a principle. Any potential connection between universalism and tolerance is rather of a wholly contingent nature (e.g., Kim and Wreen 2003; Wreen 2001). Moreover, several psychological studies suggest that as a matter of fact, being a moral universalist makes one less tolerant towards disagreeing others (e.g., Goodwin and Darley 2012; Wright et al. 2013; Wright and Pölzler forthcoming). If a universalist judges that self-driving cars ought to kill the adult who is crossing the street on a “go ahead” signal then they are hence, other things being equal, more likely to try to change the moral judgements of people who disagree or to interfere with these people’s behavior than a relativist.

So far I have argued that the importance of law and potential commitments to tolerance do not give us reason to doubt the universalism/relativism debate’s relevance for the ethics of self-driving cars. Let me now make a positive case for this relevance; in particular, by explaining in more detail *how* this debate is relevant.

To begin with, the moral universalism/relativism debate matters for the justification of moral judgements about accidents with self-driving cars. If morality were universal, and hence metaphysically invariant with regard to the moral beliefs of societies or cultures, then judgements such as “Self-driving cars ought to kill the boy who crossed the street despite the ‘do not cross’ signal” would have to be justified independently of these beliefs (e.g., by the utilitarian, contractualist or deontological arguments referenced in the introduction).^8^ Relativistic justifications, in contrast, are fully grounded in claims about societies’ or cultures’ moral beliefs. In their case, to establish that self-driving cars ought to kill the boy one would have to show that most people in one’s society or culture believe that these cars ought to kill the boy.

It is hard to deny that the universalism/relativism debate affects the nature of moral justifications in the above way, and that these justifications are relevant to applied ethics. However, critics may object that in practice it is not so relevant *why* self-driving cars are programmed to behave in certain ways; what is relevant are only *the ways in which* they are programmed to behave, i.e., the *content* of our true or justified moral judgements about this matter. Given that universalism and relativism are metaethical claims, the worry arises that at least they cannot possibly have any implications for the content of moral judgements about accidents with self-driving cars.

In response to this worry it is first worth emphasizing that the nature of moral justifications — especially of public moral justifications — is more practically relevant than suggested. For example, how certain accident behaviors are justified will likely affect the extent to which customers buy and accept self-driving cars and the ways in which governments legally regulate them. Even more importantly, in conjunction with certain empirical claims, universalism and relativism can have differing implications for the *content* of our moral judgements as well.^9^ With regard to any moral issue this is the case if the following empirical condition holds: cultures or societies differ in their dominant moral beliefs about this issue.

To see why differing moral beliefs necessarily lead universalists and relativists to accept differing moral judgements, imagine that the above empirical condition holds: culture A dominantly believes that action X is morally right, while culture B dominantly believes that action X is morally wrong. Under these circumstances relativism entails that X is morally right for the members of A but morally wrong for the members of B. Universalism, by contrast, is inconsistent with this conclusion. It commits us to holding that X is either right for both A and B or that it is wrong for both A and B (because, according to universalism, actions exemplify their moral properties independently of the moral beliefs of societies or cultures). In whatever way A and B differ in their dominant moral beliefs, then, this difference implies differing moral judgements from universalist versus relativist perspectives.

These considerations show that universalism and relativism necessarily differ in their substantive implications for the ethics of accidents with self-driving cars if cultures or societies hold different moral beliefs about this matter. So do cultures or societies hold different moral beliefs about this matter? The available empirical evidence about moral beliefs about accidents with self-driving cars is sparse and preliminary (see Sec. 5). Nevertheless, there are at least some reasons to believe that in this case the “differing moral beliefs” condition is probably fulfilled. Most importantly, this is suggested by direct evidence about the moral beliefs of cultures or societies about self-driving cars, as gathered by a large-scale psychology study entitled the “Moral Machine” project (Awad et al. 2018) as well as by research into the basic moral principles and values of different cultures and societies.

First, consider the Moral Machine study. In this study millions of subjects from all around the world were presented with scenarios (including the boy/adult scenario) in which deadly accidents with self-driving cars are unavoidable. They were then asked how self-driving cars should behave in these scenarios. The results from this study suggest that some moral preferences with regard to self-driving cars are widely shared. However, many significant cultural differences emerged as well (Awad et al. 2018). These differences were especially pronounced across three distinct “clusters”: (1) North America and many European countries (the “Western cluster”), (2) many far Eastern Countries (the “Eastern cluster”), and (3) Latin America and countries with French influence (the “Southern cluster”).

The most important differences across these clusters concern the weight that subjects attributed to certain moral preferences. First, subjects from the Eastern cluster attributed less weight to sparing younger than older people than subjects from the other clusters. Second, subjects from the Eastern cluster also attributed less weight to sparing high status people. And third, subjects from the Southern cluster attributed less weight to sparing humans rather than pets, and more weight to sparing women and fit persons.

That societies or cultures differ in their moral assessments of self-driving cars is also suggested by the fact that different societies or cultures appear to be drawn towards different general moral principles or values. Take Haidt et al.’s famous moral foundations theory (Haidt 2012; Haidt and Björklund 2008; Haidt and Joseph 2007). According to this theory, humans are naturally equipped with six innate moral modules.^10^ Their cultural surroundings then lead to differential developmental manifestations of these modules. For example, while many people in the West predominantly ground their moral judgements in the values of care, fairness and liberty, other cultures also put strong emphasis on loyalty, authority, and purity. These basic evaluative differences most likely influence people’s judgements about how self-driving cars ought to be programmed (e.g., with regard to the question of whether these cars should give preference to high-status individuals).

In summary, despite worries from law and tolerance, the moral universalism/relativism debate appears to have significant practical relevance for the issue of accidents with self-driving cars. It has straightforward implications for the justification of our judgements in this area. And, even more importantly, as different societies and cultures have different moral beliefs about accident algorithms, whether one assumes universalism or relativism also affects the content of one’s moral judgements about this issue.

## The Plausibility of Relativism

In the last section I argued that the universalism/relativism debate is practically relevant for the issue of accidents with self-driving cars. This does not yet establish, however, that the implications of relativism in fact merit consideration. It may be argued instead that relativism is philosophically implausible, and that ethicists have hence been right to neglect it when they were thinking about self-driving cars. My aim in this section is to cast some preliminary doubt on this supposition.

Relativism has been subject to a broad variety of objections. For example, it has been claimed to have obviously immoral consequences, to fare badly in explaining why people (cross-culturally) morally disagree with each other (e.g., Brink 1989), and to be unable to account for morality’s normativity (e.g., Boghossian 2011). Here I will not recapitulate all of these objections or present defenses against them — though I will touch upon some of them later (see Sec. 5 and fn. 12); and, unsurprisingly, I believe that there indeed are at least somewhat plausible defenses against all of them (see, e.g., Gowans 2015; Khoo and Knobe 2018). My plan is rather to support relativism’s plausibility by presenting a preliminary positive case in its favor; in particular, by sketching two arguments: the argument from moral diversity, and the argument from folk metaethics.

The most well-known and powerful relativist argument is the argument from moral diversity (e.g., Fraser and Hauser 2010; Prinz 2007).^11^ Cross-cultural moral diversity seems to abound. For example, I have already mentioned that different cultures hold different views about how self-driving cars ought to be programmed, and about the values of loyalty, authority, and purity. If universalists were right in their belief that moral truths hold for everyone everywhere this would mean that in all these cases of moral diversity one of the two diverging cultures is mistaken. But this implication seems implausible. How can so many people – whole cultures – err widely about matters of morality? A much better explanation seems to be that each culture has its own moral truth. Proponents of the argument hence conclude that this relativistic understanding of morality is most likely correct.

Of course, for this argument to be convincing it would need to be supplemented in several ways. Cases of apparent cross-cultural moral diversity must be shown to be genuinely moral, i.e., not fully explicable by underlying non-moral diversity (e.g., in the way in which many differences in beliefs about the permissibility of abortion may reduce to different non-moral beliefs about whether fetuses can feel pain or whether God forbids having abortions; see Brink 1989; Boyd 1988). Proponents of the argument also need to refute so called “defusing explanations” that purport to reconcile widespread moral mistakes with the existence of universal moral truths (pointing out, for example, that cultures fail to grasp these truths because they are irrational or partial with regard to it; see, e.g., Boyd 1988; Huemer 2005). Finally, relativists need to show that their explanation of widespread moral diversity is better than that of proponents of another version of non-universalism, namely nihilism (the view that moral truths do not exist at all; Mackie [1977] 2005).^12^

There is some initial philosophical and empirical work which suggests that these challenges to the argument from diversity might be met (e.g., Doris and Plakias 2008; Fraser and Hauser 2012). Below I will also mention one additional reason to believe that relativism may be more well-grounded than nihilism. This will already be in the context of my second (related) preliminary argument for moral relativism, the “argument from folk metaethics” (see, e.g., Beebe forthcoming; Pölzler and Wright forthcoming), which I will address now.

There are good reasons to believe that conceptual analyses must account for lay people’s intuitions; otherwise such analyses would not be relevant to the lives of these people and philosophy would run the risk of operating in some sort of bubble (e.g., Jackson 1998; Kauppinen 2007; Machery 2017). In the past lay people were mainly assumed to be intuitively drawn towards universalism. However, recent empirical research contradicts this claim.^13^ For example, in a study by Sarkissian et al. (2011), the more the cultural distance between disagreeing parties was made salient to subjects the less universalist their interpretations of these disagreements became. Pölzler and Wright (2020) found that many more of their subjects believed that in cases of moral disagreement both parties can be right (as entailed by relativism) rather than only one party (as entailed by universalism) or no party (as entailed by nihilism). Since the majority of lay people seem to conceive of moral judgements as relative, and since conceptual analyses must account for these intuitions, we have a *pro tanto* reason to think that relativistic analyses are to be preferred over both universalist or nihilist ones.

Again, this argument is subject to a number of challenges, such as criticisms of the evidentiary status and weight of intuitions for conceptual analysis or of the methods of the empirical research that I have referenced. For plausible defenses I direct the reader to, for example, Beebe forthcoming; Loeb 2008; Pölzler and Wright 2020, forthcoming.

The above considerations show that while there are plausible arguments against moral relativism, there are also plausible arguments for it. This is of course not meant to suggest that relativism is true — in fact, I will suggest below that when it comes to the universalism/relativism debate we rather face a situation of metaethical uncertainty. I do believe, however, that in light of the above considerations relativism should at least be taken seriously. The idea of a self-driving car that is programmed under the assumption of relativism should thus no longer be ignored either. Just as ethicists have thought about the nature, advantages and disadvantages of universalist cars, they need to think about the nature, advantages and disadvantages of the relativistic car too. This is what I will turn to now.

## The Nature of the Relativistic Car

For the moment, let us assume that in programming self-driving cars all that we consider and aim for is their being programmed to behave ethically. That is, we abstract from any non-moral reasons that might speak for or against certain accident algorithms, as well as from questions about the feasibility of these algorithms. How would self-driving cars under these assumptions — i.e., fully or paradigmatically relativistic cars — behave in accident scenarios? For example, would they kill the boy who crosses the street despite the “do not cross signal” or the adult who crosses on the “go ahead” signal?

Above I have already explained that answering these questions requires empirical data. In particular, it requires data about people’s moral beliefs about how self-driving cars ought to be programmed, or data about general moral principles and values which allow inferences to these beliefs. This is because according to relativism, the moral adequacy of accident algorithms is fully determined by the moral beliefs held in a society or culture.

Suppose, for example, we find a dominant belief in culture A that killing the boy in our scenario is right, whereas the dominant belief in culture B is that killing the adult is right. According to universalist theories, this difference does not matter (much). Self-driving cars should behave in (roughly) the same way wherever they are driving, within the constraints set by local laws.^14^ Assuming relativism, however, self-driving cars would have to be programmed in such a way that they reflect this cross-cultural moral difference. They should kill the boy as long as they are on the territory of culture A (as in this situation the members of culture A are the relevant appraisers), and the adult as long as they are on the territory of culture B (as in this situation the members of culture B are the relevant appraisers).

The above example may strike you as abstract and hypothetical. However, its underlying empirical assumption may actually not be too far from reality. In Sec. 1 I explained that, according to the Moral Machine project, people from the Eastern cluster attribute less weight to sparing younger than older people than people from the Western and Southern clusters (Awad et al. 2018). Suppose this is true. Then a relativistic car that crossed the border from Russia (which is part of the Western cluster) to China (which it part of the Eastern cluster) should give less weight to sparing the boy in our scenario than it did before. If the car crossed the border in the other direction — from China to Russia — the weight it gives to sparing the boy should increase.^15^

Let me also briefly state what relativism in the sense that is at issue here does *not* imply for programming self-driving cars. First, under the assumption of relativism it is not the case that every accident algorithm that any individual believes to be morally right is in fact morally right, and that hence each driver should have his or her own algorithm (see, e.g., Contissa et al. 2017 who argue in favor of an “ethical knob”^16^; and Millar 2014). Second, the relativistic car does not need to reflect the dominant traditions, practices or sentiments of a society or culture (where these traditions, practices or sentiments do not (fully) correspond with its dominant moral beliefs). And third, what determines accident algorithms’ programming are not the moral beliefs of the manufacturer’s society or culture, but only the beliefs of the society or culture in which the self-driving car is currently operating. These operating societies or cultures are the relevant appraisers because they are most strongly affected by how the self-driving cars are programmed (boys or adults in these societies or cultures, and not in the societies or cultures of the manufacturers, will live or die depending on the programs).^17^

At first sight, then, the nature of the relativistic car seems quite simple and straightforward. It may be summed up by the proverb “When in Rome do as the Romans do” (bearing in mind, of course, that strictly speaking the self-driving car should do as the Romans *believe*). If one considers the idea more closely, however, one finds that in many situations the behavior of relativistic cars will depend on rather intricate philosophical questions about how to understand relativism more precisely. Recall that according to my generic definition, relativism is the claim that an action has a moral quality if and only if it is believed to have this quality by the majority of the members of one’s society or culture. This leaves open, among others, (1) what is meant by a *society* or *culture*, (2) what is meant by a group of people within some society or culture constituting a *majority*, (3) what is meant by a moral belief being *moral*, and (4) what is meant by a moral belief being a *belief*.

As to the first ambiguity, societies or cultures can be defined very broadly (such as in the sense of the Moral Machine project’s cultural clusters, which span many countries and even world regions) but also quite narrowly (allowing, for example, to speak of a South Carolinian or Bavarian culture). One’s preferred understanding of these terms significantly affects how often and in what ways relativistic cars’ accident algorithms will need to switch from one moral setting to another. For example, on the cultural clusters account, accident algorithms would only have to involve two settings regarding the moral weight of sparing the young, one for the Eastern cluster and one for Non-Eastern clusters. If one defines cultures in terms of smaller societal units, in contrast, self-driving cars may need to weigh sparing the young in dozens, hundreds or maybe even thousands of different ways in order to behave ethically.

Suppose relativists have settled on a particular understanding of “culture or society”. Another challenge arises from the fact that such units typically are not homogenous but also involve a certain amount of diversity within themselves. This means that proponents of the relativistic car also owe us an account of what it means for a particular moral belief to be held by a “majority” of people in a culture or society. For example, does a simple majority suffice? Or should we rather go for some form of qualified majority? What if the members of a culture or society hold potentially incommensurable values (such as efficiency and safety with regard to speed limits) which entail incoherent judgements about accident scenarios? Etc.

An intricate problem with defining the notion of a moral belief is that the boundaries of the moral domain are notoriously contested (see Machery 2012). For example, do moral beliefs entail categorical demands? Are they intrinsically motivating? Are they necessarily about harms and benefits? It is therefore often unclear whether by making some statement a person intends to express a belief about morality or rather about her personal preferences, social conventions or other non-moral facts. According to relativism, only people’s *moral* beliefs determine the rightness, wrongness, goodness, badness, etc. of an action. Proponents of this view therefore owe us criteria for distinguishing these beliefs from non-moral beliefs.

Finally, quite different things can be understood by the term “belief” as well. On one end of the spectrum we find interpretations according to which relativists claim that moral rightness, wrongness, goodness, badness, etc. depend on people’s immediate moral gut reactions: on how things seem to them at first glance. Representing the other end of the spectrum, the relevant notion of belief may also be understood as referring to full-blown beliefs resulting from rational reflection. Moreover, to the extent that relativists plan on relying on data about general moral principles and values (such as care, fairness, liberty, etc.), they must also explain how to derive judgements about particular moral issues (such as the boy/adult scenario) from these principles and values.

Below I will occasionally come back to the above unclarities; resolving them is a central challenge for those who favor relativistic self-driving cars. For now, let us suppose we have fully figured out how a relativistic car would be programmed to behave. The obvious next question to ask is then whether self-driving cars *ought* to be programmed in this way.

At first sight the answer to this question might seem trivial. For illustrative purposes, in this section I have assumed that moral relativism is true. Under this assumption the only way to program a fully ethical car is to program a relativistic car. No other (non-relativistic) car could consistently meet morality’s requirements. Clearly, then, we ought to program cars in this way — don’t we? However, this conclusion would be hasty. In making decisions about how to program self-driving cars we should not only consider *moral* “oughts”, but also accident algorithms’ non-moral consequences (such as their prudential or legal consequences) and their feasibility (such as their technical or political feasibility).

That non-moral consequences and feasibility matter or matter much might be contested. So let me briefly defend this claim. To begin with, in contrast to some philosophers, I think that there are good reasons to believe that moral norms do not always override other kinds of norms. Under some circumstances non-moral norms may well weigh heavier (Terrell 1969; Portmore 2008). This is particularly true when there is uncertainty about what morality requires. As it happens (this will be explained in Sec. 5 below), on the basis of relativism we actually sometimes find ourselves in such a situation: we do not (fully) know how self-driving cars ought to be programmed. Finally, almost all philosophers accept the principle “ought implies can” (see Kant [1788] 2015; Vranas 2007); and many even accept that we cannot be required to do what is possible but infeasible (e.g., Farelly 2007; Gaus 2017).^18^ If a certain accident algorithm is infeasible for technological, political or other reasons I hence take this to mean that we are under no moral obligation to implement this algorithm.

There is one more reason why the non-moral consequences and feasibility of the relativistic car deserve consideration. So far I have proceeded under the assumption that relativism is true. But in the real world — the world that we should actually consider when we make decisions about self-driving cars — there is of course uncertainty about this metaethical claim. Just like relativists, universalists and nihilists can point to plausible arguments in their favor (e.g., Hare 1954; Mackie [1977] 2011; Taylor 1978), and to plausible objections against relativism (as referenced in Sec. 2). We hence do not know with anything near certainty which of these positions is the correct one. This metaethical uncertainty further increases the uncertainty at the first-order moral level; for now we also need to consider that the morally best accident algorithm might conform to any of the numerous universalist theories that have been proposed (such as utilitarianism, deontology or contractualism) or that there is no such algorithm at all (provided that nihilism is true). Under these circumstances it seems all the wiser to give significant weight to different algorithms’ (more certain) non-moral consequences and feasibility as well.

## The Advantages of the Relativistic Car

In this Section I will sketch some of the advantages of programming self-driving cars according to relativism. My focus will be in particular on what such accident algorithms would mean for establishing self-driving cars, for traffic safety, for our certainty about how these cars ought to be programmed, and for accounting for cross-cultural moral differences.

Traffic systems that involve self-driving cars will promote the self-interest of (most members of) almost any society or culture around the world. The overwhelming majority of road accidents are due to human error, such as speeding, distraction and intoxication. These new systems will hence make roads significantly safer. They have the potential to prevent tens of thousands of deaths and hundreds of thousands of injuries each year (e.g., Gogoll and Müller 2017; Hevelke and Nida-Rümelin 2015). Moreover, self-driving cars will allow physically impaired, elderly and young persons to drive; they will go at speeds that require less gas consumption, thereby decreasing greenhouse gas emissions; they will reduce traffic jams by automatically taking the fastest routes and being able to drive close after one another; and so on.^19^

Given that self-driving cars will increase overall well-being, an important criterion in evaluating accident algorithms is how these algorithms will affect self-driving cars’ widespread introduction. Algorithms that promote this introduction are, ceteris paribus, to be preferred to algorithms that hinder it (Bonnefon et al. 2015; Hevelke and Nida-Rümelin 2015). One plausible prediction in this regard is that self-driving cars will become established faster to the extent to which they conform to people’s moral beliefs. For example, a person who believes that self-driving cars should kill the adult in our above scenario will be more likely to buy a self-driving car, to approve of others buying them, to support legislation in favor of these cars, etc. if the cars are programmed to actually kill the adult. This effect will probably be even stronger in the case of moral decisions that involve persons’ own safety as drivers (as people of course prefer to be saved themselves, see Bonnefon et al. 2016).

Under the assumption of universalism the accident behavior of self-driving cars may diverge significantly from what people ordinarily regard as morally right. At the very least, this will hold for *some* societies or cultures. (Recall that universalist cars behave the same regardless of where they operate, and different societies’ and cultures’ moral beliefs about accident algorithms differ.) In contrast, consider the relativistic car. This car per definition reflects the majority’s moral beliefs within *any* society or culture. Wherever it drives, it will always behave in such a way as to best account for the prevailing ethical preferences. This suggests that self-driving cars might become established faster if they are programmed in a relativistic manner. People might be more likely to buy such cars, to approve of others buying them, to support legislation in their favor, and so on.

My above argument assumes, among others, that self-driving cars will make traffic safer. This assumption is highly plausible. Yet, *how much* safer traffic will become partly depends on the extent to which self-driving cars’ accident algorithms are coordinated. To be maximally effective in making traffic safer these cars need to behave consistently (Bonnefon et al. 2015; Gogoll and Müller 2017). For example, if some cars in a mass crash are programmed to kill a boy on one side of the road while others are programmed to kill an adult on the other side then the overall result in terms of deaths and injuries will often be worse than if all cars behave in the same way (thereby sparing at least either the boy or the adult). Universalist cars clearly fulfill this consistency condition. It is important to point out, however, that relativistic cars do so too. This is because, within any given society or culture, all relativistic cars will run on the same accident algorithm. It cannot be the case, for example, that one car is programmed to kill the boy and another car is programmed to kill the adult in the above scenario; for (leaving aside complications such as those mentioned in the last section) it cannot be the case that within any particular society or culture there is more than one dominant belief about how self-driving cars ought to behave in this situation.^20^

One main problem with programming self-driving cars under the assumption of universalism is epistemic. Normative ethics abounds with competing theories that all purport to capture the universal moral truth. Proponents of numerous versions of utilitarianism disagree with proponents of numerous versions of deontology, proponents of numerous versions of contractualism, and so on (Bourget and Chalmers 2014). Each of these theories has differing implications for the ethics of accidents with self-driving cars. Hence, given universalism, decision-makers face a situation of extreme moral uncertainty (see Bykvist 2017). They cannot be sure at all how self-driving cars morally ought to behave in this or that situation. Whatever theory or theories manufacturers may rely on, or whatever computational framework they may employ for accounting for moral uncertainty,^21^ universalism always entails a high risk of moral error or at least of non-optimal moral decisions (see Klincewicz and Frank 2018).

In the next Section I will argue that the relativistic car is prone to worries about uncertainty as well. However, this uncertainty is mainly empirical. In terms of genuinely *moral* uncertainty the relativistic car fares significantly better than its universalist counterpart (see Klincewicz and Frank 2018). Under the assumption of relativism there is a reasonably clear and uncontroversial answer to any moral question about accident algorithms. These algorithms should always be designed in such a way that they reflect the dominant moral beliefs of the society or culture that the car operates in. Hence, considering only genuinely moral uncertainty, the relativistic car involves a lower risk of making moral mistakes or non-optimal moral decisions. With the stakes as high as they tend to be in traffic (where injuries and deaths are a common sight), and people generally being highly avers to making moral mistakes and non-optimal moral decisions (so as to avoid regret, feelings of guilt, moral anxiety, etc.), this is another important advantage.

Finally, the relativistic car also fares better in accounting for cross-cultural moral differences. Self-driving cars’ accident algorithms will mainly be fixed in Western countries such as the US and Germany, as well as in Japan (as these countries will be the main manufacturers of self-driving cars). Under the assumption of universalism these cars will behave identically in all societies and cultures over the world. For example, they will either kill the boy or kill the adult in our main scenario, irrespectively of where they operate. This raises the worry of moral colonialism, i.e., of a small proportion of humanity imposing its moral views on the whole rest of the world. Azim Sharrif, one of the lead researchers in the Moral Machine project, recently explicitly raised this worry. He urged manufacturers to be “sensitive to the cultural differences in the places they’re instituting […] ethical decisions” (cited in Lester 2019).^22^

Most people in most societies and cultures are opposed to moral colonialism. If they regard a certain behavior as right then they want their technological devices, government, companies, fellow citizens etc. to proceed under the assumption that the behavior is in fact right; even if it is morally disapproved of by members of other societies or cultures. This suggests that programming self-driving cars according to universalism runs the risk of creating negative feedback effects in “colonialized” societies and cultures. It may cause public outrage; it may lead to hostile attitudes towards those countries or manufacturers that fix the algorithms of self-driving cars; and, most importantly, it may prevent some societies and cultures from introducing self-driving cars (as fast as they could) in the first place, thus forfeiting or delaying the well-being increases that these cars tend to bring about. By letting each society or culture determine its own accident algorithm the relativistic car avoids these negative effects.^23^

## The Disadvantages of the Relativistic Car

The relativistic car brings not only advantages. Some of its non-moral consequences and feasibility-related aspects also raise worries. In this Section I will introduce some of these worries, focusing on potential immoral consequences, the misuse of relativistic algorithms, and uncertainties concerning the ethical programming.

The most obvious objection against relativistic accident algorithms is that they can have what seem to be morally outrageous consequences. Suppose, for example, there is a dominant belief in a society that in cases of accidents self-driving cars should always favor white people over people of color, or men over women. Under the assumption of relativism this would mean that self-driving cars *morally ought to* be programmed to reflect these beliefs: they *ought to* always favor white people over people of color, or men over women *morally*. But accident algorithms operating on this basis would clearly appear to be morally outrageous. Hence, according to the objection, the idea of the relativistic car must be rejected.

Relativists have replied to this immorality objection in a number of ways (for discussion see, e.g., Gowans 2015; Westacott 2012). Most plausibly, they have incorporated at least some very broad universality constraints. According to Wong (2006), for example, what is morally right, wrong, good, bad, etc. is determined by the moral beliefs of one’s society or culture; but only as long as it does not violate certain rules that are grounded in human nature and the human condition. For example, this could mean that while self-driving cars morally ought to kill the adult who crosses the street on the “Go Ahead” signal in some societies, they morally ought to kill the boy who crosses the street on the “Do not Cross” signal in other societies; but that regardless of people’s moral beliefs it can never be right for these cars to be programmed to always favor men or white people.

To me, while still holding on to my claims about metaethical uncertainty, such qualified versions of moral relativism seem most plausible. But I would also like to raise awareness for a different fact about the above objection. The immorality objection does not target the relativistic car’s non-moral consequences or feasibility. It is rather about its philosophical soundness. This means that the preceding discussion actually belongs in Section 2 rather than in the present Section, i.e., it concerns whether relativism is plausible. If we interpret the objection as a worry about bad *non-moral* consequences, in contrast, its weight seems to be relatively low. For example, in the above hypothetical scenario racist or sexist accident algorithms are widely accepted within the relevant society, which means that they will not hinder the introduction of self-driving cars; in the real world relatively few societies may have a moral preference for (strongly) racist or sexist algorithms (e.g., in the Moral Machine study participants from *all* cultural clusters on average tended to favor female over male road users; Awad et al. 2018); and where relativism implies discriminatory algorithms after all such tendencies will sometimes be mitigated by state or human rights law.

A more serious worry about the relativistic car is that it might facilitate misuse by powerful individuals or institutions. In a recent interview Udo Di Fabio, a former constitutional judge and leader of a German commission on the ethics of self-driving cars, expressed concerns about the possibility of China programming cars so that they spare people who score high in their social-credit system (a system that rewards civic and penalizes uncivic behavior) (cited in Lester 2019). One might also be concerned about rich individuals bribing government officials or car manufacturers so as to increase their own road safety or the safety of their families or friends. Any such misuse would go against the interests of the majority of any society’s or culture’s members.

Proponents of the relativistic car might respond that universalism does not (fully) prevent misuse either. Even if self-driving cars are supposed to run on the same accident algorithm always and everywhere, this algorithm can still be tinkered with. However, while this observation is correct, relativism at least *increases* the risk of misuse. The accident algorithms of relativistic cars will often change, so as to account for changes in moral beliefs. It will thus be harder for people to identify illegitimate behavioral manifestations of these algorithms, and easier for manipulators to implement their preferred changes (at regular updates). Moreover, the behavior of relativistic cars will mostly have to be determined and justified by empirical studies or referenda. These studies or referenda can be manipulated more easily than the normative ethical arguments of universalists. For example, subtle changes in the formulation of scenarios may lead subjects to respond differently than they would otherwise have done; only specific populations may be surveyed; and governments or companies may exclude unwanted results or fabricate data.^24^

This leads to another intricacy of the relativistic car. In the last Section I argued that in contrast to their universalist counterparts, such cars do not involve much genuinely moral uncertainty. But even under the assumption of relativism we will sometimes be unable to tell (precisely) how self-driving cars ought morally to behave in specific situations. This is because, as just hinted at, one needs empirical data in order to determine the accident algorithms of relativistic cars: data about people’s moral beliefs about how self-driving cars ought to be programmed, or data about general moral principles and values which allow inferences to these beliefs. Valid data of this kind — especially of the first (presumably more relevant) kind — is difficult to obtain.

One main source of difficulty in gathering relevant data is that in the sense in which relativism has been discussed here (and in many other places) it is unclear. As explained in Sec. 3, my definition left open, among other things, what is meant by a “society” or “culture”, what is meant by a moral belief being “moral”, and what is meant by a moral belief being a “belief.” We will only be in a position to know what kind of empirical evidence is needed to specify relativistic accident algorithms once all of these terms have been defined in sufficient detail. Any definitions of the terms “society”, “culture”, “moral” and “belief”, however, are bound to be philosophically controversial. This means that studies on or referenda about people’s beliefs about the ethics of accidents with self-driving cars will, at least to some extent, always be open to reasonable conceptual debate.

In addition, there are also several methodological and pragmatic challenges in gaining the evidence that would be required for specifying relativistic accident algorithms. Let me provide but three examples. First, studies on the beliefs people have about the ethics of accidents with self-driving cars need to be based on representative samples. For instance, collecting only the responses of internet volunteers (as in the Moral Machine study) might bias the results. Second, as suggested above, studies and referenda need to be repeated at short intervals, so as to account for changes in moral beliefs. Such changes are particularly likely to occur during the early stages of the introduction of self-driving cars (see Nyholm 2018a). And third, studies and referenda also need to be ecologically valid, i.e., their materials and procedures need to be such that they allow generalizations to real life. This may, among others, speak against presenting only life/death scenarios (as in the Moral Machine study). For one thing, subjects may not have clear intuitions about these extreme cases. For another thing, their responses may be affected by their perceiving these cases as unrealistic or possibly even humorous (see Bauman et al. 2014).

To be sure, some versions of the universalist car suffer from empirical in addition to moral uncertainty too. An act utilitarian accident algorithm, for example, requires input about the consequences of each of the car’s potential behaviors in each situation. This input can never be fully provided either. With many universalist moral theories, however, the empirical (not moral) uncertainty faced by universalist cars is lower than that of relativist cars.

## Conclusion

This paper addressed the relevance and implications of moral relativism for the ethics of accidents with self-driving cars. I began by arguing that whether one adopts a universalist or a relativist perspective with regard to this ethical question matters, and that the relativist perspective must not be dismissed easily. I then went on to consider the nature of the relativistic car and its advantages and disadvantages. To reemphasize, these considerations were of an exploratory, preliminary, and incomplete nature. They provided some initial evidence that compared to universalist cars, relativistic ones would have several important advantages in terms of non-moral value and feasibility. I did not explicitly weigh these advantages against relativistic cars' disadvantages. However, at least on the face of it, it seems that in the considered respects relativistic cars may be preferable to universalist ones.

Note that the point of this paper was to provide a first explicit discussion of the idea of the relativistic car. I did not mean to endorse this car. As mentioned in Sec. 3, I consider the current epistemological state with regard to the universalism/relativism debate to be one of uncertainty. We cannot be sure whether and in what sense morality is universal or relative. In light of the available evidence, I take it that either of these views could be true. As mentioned in Sec. 5, it might even be most plausible to combine relativism with certain elements of universalism, such as in the theories of many contemporary relativists or functionalists (e.g., Wong 2006). Finally, it also turned out that even though the relativistic car seems preferable in terms of non-moral consequences and feasibility, it is far from flawless in these respects either.

In any case, I hope that my considerations in this paper will initiate a broader discussion about metaethics’ relevance and implications for moral questions relating to self-driving cars. Infusing these cars with (at least some) relativism appears to be an idea worthy of further exploration.

## Footnotes

1 By self-driving cars I here mean cars that operate on very high levels of automation, where these cars’ algorithms make all or most decisions without human intervention.

2 Basl and Behrends (2019) argue that the behavior of self-driving cars will not be governed by traditional but by machine-learning algorithms, which means that this behavior will also (in addition to software engineers’ decisions) be determined by learning processes. As a consequence, in actual practice, the programming of self-driving cars might be a less straightforward matter than is suggested in this paper.

3 To my knowledge, the implications of metaethics for decision-making in artificial intelligence have so far only been investigated in detail by Klincewicz and Frank (2018). However, their paper does not focus on the universalism/relativism debate (but rather on the realism/anti-realism debate), and does not relate metaethics to the specific issue of accidents with self-driving cars (but rather to artificial intelligence in general).

4 Participants of the debate have not *explicitly* stated that they start from universalism. However, as indicated above, they typically have not relativized their ethical conclusions in any way (conceding, for example, that they are valid within North America but not within some other cultures). Moreover, the moral principles that they have appealed to (consequentialism, deontology, etc.) are typically supposed to apply universally. This indicates that even if only implicitly the debate about accidents with self-driving cars is dominantly committed to moral universalism.

5 A small minority of philosophers believe that metaethical claims such as universalism and relativism are actually only first-order moral claims in disguise (e.g., Dworkin 1996). Here I will proceed under the assumption that this “meta-metaethical” view is mistaken (see also fn. 9 below).

6 It also bears mentioning that to the extent that a country’s traffic laws reflect its dominant moral beliefs, self-driving cars will be relativistic by default.

7 This taxi service by Waymo, an Alphabet (Google) company, does not require any safety driver (Ohnsman 2019).

8 Note that some universalist theories can account for societies’ or cultures’ moral beliefs. For example, given that people may have a preference for self-driving cars behaving according to the moral beliefs of their society or culture, preference-utilitarians may include them in their calculus. Even in these cases, however, moral beliefs are not what *makes* moral judgements true – and hence, these judgements are still invariant with regard to these beliefs in a metaphysical sense.

9 The above argument assumes that universalism and relativism, even though metaethical claims, have at least some normative content. This is for the following reason. Hume’s Law claims (very plausibly) that no set of exclusively descriptive premises can entail a normative conclusion. Thus, if universalism and relativism where exclusively descriptive then it would not be possible for them to entail any first-order moral judgements in conjunction with (exclusively descriptive) empirical claims, be these judgements about accidents with self-driving cars or about any other matter (see Dworkin 1996). In contrast to Dworkin and others, however, I do not believe that this fact forces us to regard universalism and relativism as first-order moral judgements in disguise. Their descriptive philosophical commitments still significantly distinguish them from ordinary first-order moral judgements (see Kagan 1998; McPherson 2008).

10 Initially moral foundations theorists endorsed only five of these modules (Haidt and Björklund 2008; Haidt and Joseph 2007). The sixth (liberty) was suggested in a more recent book by Haidt (2012).

11 As mentioned above, in support of their view universalists have sometimes appealed to moral disagreement as well, e.g., by claiming that the existence of genuine moral disagreement is inconsistent with relativism or nihilism (e.g., Strandberg 2004) or that the fact that people disagree about moral propositions suggests that they are implicitly committed to universalism, which in turn is claimed to support universalism (e.g., Brink 1989). These arguments do not strike me as convincing. Attempts to refute them can be found, e.g., in Loeb 2007; Pölzler 2018; for discussion see Francén 2016.

12 This metaethical position — moral nihilism — was actually the conclusion that the argument from moral diversity is supposed to support in its most famous formulation by Mackie ([1977] 2011).

13 This conclusion seems to conflict with the results of earlier studies on folk metaethics (e.g., Goodwin and Darley 2008). However, properly interpreted, even these earlier studies do not support a tendency towards universalism (e.g., Pölzler 2017).

14 Again, as acknowledged in fn. 9, some universalist theories account for societies’ or cultures’ moral beliefs to at least some extent and in some sense.

15 This is perhaps the most natural way in which cross-border driving could be handled. Some of the programming of self-driving cars must be adapted to particular countries anyhow, to comply with these countries’ laws. Going morally relativistic would just mean that these cars’ algorithms must change more strongly when they cross the borders of countries or cultures — which does not seem technologically or financially infeasible. That said, the issue of cross-border traveling is an intricate one which requires more discussion than I can provide here.

16 By an „ethical knob“Contissa et al. mean „a device enabling passengers to ethically customise their AVs [autonomous vehicles], namely, to choose between different settings corresponding to different moral approaches or principles“(2017: 365).

17 It is of course also possible for members of the manufacturer’s culture to make moral judgements about self-driving cars in other cultures. In this case, these judgements are made true by what the majority of members in the manufacturer’s culture believe. If the manufacturer’s culture would dominantly kill the adult, members of this culture may hence rightfully morally criticize the operating culture for opting to kill to boy. But this criticism would always have to be levelled in a way that makes it clear that it comes from within the manufacturer’s culture, and does not apply from the perspective of the operating culture; for according to moral relativism, no particular moral framework can be claimed to be superior to any other (Kölbel 2015; Westacott 2012).

18 In political philosophy, those who endorse strong feasibility requirements are typically referred to as proponents of „non-ideal“(as opposed to „ideal“) theory. In this paper I endorse such a non-ideal approach, as my aim is to inform how self-driving cars ought to be programmed here and now, given the circumstances that we find ourselves in.

19 There are of course also prudential reasons *against* introducing self-driving cars. However, participants of the debate widely and plausibly agree that these reasons are clearly outweighed by reasons in self-driving cars’ favor.

20 By entailing coordinated accident algorithms the relativistic car is at an advantage over cars that are programmed to conform to each driver’s individual moral beliefs, such as with the “ethical knob” proposal mentioned in Sec. 3.

21 For moral uncertainty frameworks that have been proposed in the context of discussions about the ethics of self-driving cars see, e.g., Bhargava and Kim 2017; Bogosian 2017.

22 One might object that the relativistic car at least does not account for *intra*-cultural moral differences. After all, even within a culture the moral beliefs of individuals and groups tend to differ. This is of course correct. However, relativizing moral truth to individuals has so many disadvantages vis-a-vis relativizing it to societies or cultures (some of which have already been hinted at, such as exacerbating the coordination of accident algorithms) that this advantage is easily outweighed.

23 In response to this objection, universalists might reply that their view can be combined with a principle of tolerance. I have already raised worries about this reply in Section 1.

24 At the very least, the above suggests that with the relativistic car it would be important to have accident algorithms determined and supervised by some sort of intergovernmental organization.
